# The Arabidopsis KASH protein SINE3 is involved in male and female gametogenesis

**DOI:** 10.1007/s00497-024-00508-8

**Published:** 2024-09-16

**Authors:** Morgan Moser, Norman R. Groves, Iris Meier

**Affiliations:** 1 Department of Molecular Genetics, The Ohio State University, Columbus, OH USA; 2 Center for RNA Biology, The Ohio State University, Columbus, OH USA; 3 Present Address: Institute of Genomic Medicine, Nationwide Children’s Hospital, Columbus, OH USA

**Keywords:** LINC complex, KASH protein, Female gametophyte, Male gametophyte, Nuclear envelope, Pollen mitosis 1

## Abstract

**Key message:**

The Arabidopsis KASH protein SINE3 is involved in male and female gametophyte development, likely affecting the first post-meiotic mitosis in both cases, and is required for full seed set.

**Abstract:**

Linker of nucleoskeleton and cytoskeleton (LINC) complexes are protein complexes spanning the inner and outer membranes of the nuclear envelope (NE) and are key players in nuclear movement and positioning. Through their roles in nuclear movement and cytoskeletal reorganization, plant LINC complexes affect processes as diverse as pollen tube rupture and stomatal development and function. KASH proteins are the outer nuclear membrane component of the LINC complex, with conserved C-termini but divergent N-terminal cytoplasmic domains. Of the known Arabidopsis KASH proteins, SUN-INTERACTING NUCLEAR ENVELOPE PROTEIN 3 (SINE3) has not been functionally characterized. Here, we show that SINE3 is expressed at all stages of male and female gametophyte development. It is located at the NE in male and female gametophytes. Loss of SINE3 results in a female-derived seed set defect, with *sine3* mutant ovules arresting at stage FG1. Pollen viability is also significantly reduced, with microspores arresting prior to pollen mitosis I. In addition*, sine3* mutants have a minor male meiosis defect, with some tetrads containing more than four spores. Together, these results demonstrate that the KASH protein SINE3 plays a crucial role in male and female gametophyte development, likely affecting the first post-meiotic nuclear division in both cases.

**Supplementary Information:**

The online version contains supplementary material available at 10.1007/s00497-024-00508-8.

## Introduction

Sexual reproduction requires the production of haploid gametes that fuse to form a diploid zygote. Reproduction in flowering plants has evolved to use pollen tubes to deliver sperm cells to female gametes (Johnson et al. 2019). Shortly after pollination, the pollen grain is hydrated. The pollen tube penetrates the pistil and grows through the transmitting tract towards the female gametophyte, or embryo sac (Palanivelu and Tsukamoto 2011). Upon arrival at the ovule, the pollen tube is guided towards the micropylar opening where it contacts one of the two synergid cells of the embryo sac (Dresselhaus et al. 2016). The pollen tube ultimately ruptures and releases the sperm cells for fertilization (Dresselhaus et al. 2016).

Plants have a two-staged life cycle in which a haploid gametophyte generation alternates with a diploid sporophyte generation. Flowering plants have male and female multi-cellular haploid gametophytes. Haploid cells in the gametophyte undergo two to three rounds of post-meiotic mitosis, to form gametes and the accessory cells required for reproduction (Drews and Yadegari 2002). During male gametogenesis, the diploid pollen mother cell (PMC) undergoes meiosis and produces a tetrad of haploid microspores encased in callose (Twell et al. 1998; Twell 2011; Oh et al. 2011). Once released from the tetrad, the microspores grow in size and the nucleus migrates to the germ cell pole (Twell et al. 1998; Twell 2011; Oh et al. 2011). The polarized microspore undergoes an asymmetric mitotic division, termed pollen mitosis I (PMI), which produces a generative cell within the vegetative cell (Twell et al. 1998; Twell 2011; Oh et al. 2011). The generative cell then undergoes a second mitosis (PMII) to form two identical sperm cells.

During female gametogenesis, the megaspore mother cell (MMC) undergoes meiosis, resulting in four megaspores. Subsequently, three megaspores degenerate, leaving one functional megaspore (FM) (Erbasol Serbes et al. 2019). The FM then undergoes three rounds of mitosis without cytokinesis (Erbasol Serbes et al. 2019). After subsequent cellularization and polar nuclei fusion, the FM becomes a seven-celled female gametophyte, containing an egg cell, two synergid cells, a diploid central cell, and three antipodal cells, which undergo cell death before fertilization (Erbasol Serbes et al. 2019).

Similar to animals, flowering plants undergo open meiosis and mitosis. This involves breakdown of the nuclear envelope (NE), allowing for the connection of the kinetochores to the spindle fibers and for chromosome separation. The NE then reforms upon completion of nuclear division (Pradillo et al. 2019). The proteins of nucleoplasm and cytoplasm that interact with the NE are crucial for the successful completion of mitosis and meiosis (Pradillo et al. 2019). The linker of nucleoskeleton and cytoskeleton (LINC) complex is an important player in various protein interactions at the NE. LINC complexes are embedded in the NE and are composed of outer NE Klarsicht/ANC-1/Syne Homology (KASH) proteins and inner NE Sad1/UNC-84 (SUN) proteins that interact in the NE lumen. The terminal four amino acids of KASH proteins interact with the C-terminal SUN domain of the SUN proteins to form a bridge between the nucleoplasm and the cytoplasm (Starr and Fridolfsson 2010; Zhou et al. 2014).

Animals and plants have homologous SUN proteins (Graumann et al. 2010; Oda and Fukuda 2011). The outer NE KASH proteins appear to have evolved separately in plants, because they have nothing other in common with animal KASH proteins than being tail-anchored proteins with a highly conserved, immediately C-terminal short amino acid sequence (Xu et al. 2007; Zhou et al. 2012, 2014). Several plant KASH proteins have been functionally investigated in Arabidopsis, and are involved in a variety of tissues and processes (Meier et al. 2017). The plant KASH proteins WIP1-WIP3, along with their outer nuclear envelope interaction partners WIT1 and WIT2, are involved in male fertility and the movement of the pollen nucleus (Zhao et al. 2008; Zhou and Meier 2014; Zhou et al. 2015b; Moser et al. 2020) and shape and movement of the root hair nucleus (Zhou et al. 2012, 2015a; Tamura et al. 2013). The plant KASH proteins SINE1 and SINE2 are involved in stomatal development and stomatal dynamics (Gumber et al. 2019; Biel et al. 2020a; Biel et al. 2020b; Biel et al. 2022; Biel and Moser et al. 2024). In the model legume *Medicago truncatula*, plant KASH proteins are involved in initiation of nodulation (Newman-Griffis et al. 2019). All identified Arabidopsis KASH proteins bind to the INM-localized SUN proteins SUN1 and SUN2 (Zhou et al. 2012, 2014). The underlying assumption is that all these roles are, in analogy to the function of animal KASH proteins, related to movement or positioning of the nucleus or chromatin organization in different cellular situations.

SUN-BINDING NUCLEAR ENVELOPE PROTEIN 3 (SINE3) was identified by its plant KASH C-terminus and shown to be associated with the Arabidopsis nuclear envelope in a SUN-dependent manner, but has not been functionally investigated (Zhou et al. 2014). Here, we show that Arabidopsis SINE3 plays a role in gametophyte development. SINE3 is located at the NE in developing male and female gametophytes. Loss of SINE3 results in reduction in seed set and silique length, that is driven by defects in female gametophyte development, as *sine3* mutant ovules arrest prior to the first post-meiotic mitosis. Pollen viability is significantly reduced in *sine3* mutants as well, as a result of microspores arresting prior to the first post-meiotic mitosis during male gametophyte development. Together, these data indicate that SINE3 is involved in both male and female gametogenesis in Arabidopsis, and likely has a role in the first post-meiotic nuclear division.

## Materials and methods

### Plant material and growth

*Arabidopsis thaliana* (Columbia-0 ecotype) was germinated on Murashige and Skoog (MS) medium plates (Caisson Laboratories) containing 1% sucrose under constant light. Plants at the two-leaf stage were transplanted to soil and grown at an average temperature of 22–23 °C under a 16-h light/8-h dark regime. *sine3-1* (SALK_032814C), *sine3-2* (SALK_029812), and *sine3-3* (SAIL_248_C12) were obtained from the Arabidopsis Biological Resource Center (Alonso et al. 2003; Sessions et al. 2002). The primers used to genotype the T-DNA insertion mutants are listed in Supplemental Table 1.

### Cloning

The SINE3 promoter was amplified from whole seedling genomic DNA (~ 2.2 kb; primers used are listed in Supplemental Table 2). Restriction sites for enzymes *SacI* and *SpeI* were added to the 5’ and 3’ ends and the amplified fragment was digested with the appropriate restriction enzymes. The SINE3 promoter fragment (approximately 2200 basepairs upstream of the SINE3 start codon) was isolated and purified with the QIAquik PCR Purification kit (Qiagen). The isolated fragment was subsequently ligated into a pH7WGF2 Gateway vector to obtain SINE3pro@pH7WGF2 (Takagi et al. 2011). PCR-based cloning was used previously to generate β-glucuronidase (GUS) and SINE3 coding regions, which were cloned into pENTR/D-TOPO vectors (Zhou et al. 2014). By LR reaction, the GUS, SINE3, and SINE3ΔPLPT sequences were moved from the pENTR/D-TOPO to SINE3pro@pH7WGF2 to obtain the SINE3pro::GFP-GUS, SINE3pro::GFP-SINE3, and SINE3pro::GFP-SINE3ΔPLPT constructs, respectively.

### Generation of transgenic plants expressing GFP-tagged constructs

Binary vectors were transformed into *Agrobacterium tumefaciens* strain ABI by triparental mating (Wise et al. 2006). The Agrobacterium-mediated floral dip method was used to transform either Col-0 ecotype (WT) or *sine3-1* (Clough and Bent 1998). Transgenic plants were isolated on MS plates supplemented with 30 µg/mL hygromycin, and the positive transformants (T_1_ plants) were confirmed by using confocal microscopy to detect GFP fluorescence. Each T_1_ transgenic plant was a result of an independent insertional event of the T-DNA of interest within the Arabidopsis genome. Progeny from each T_1_ plant were grown on hygromycin selection and floral tissue was imaged to confirm presence of GFP fluorescence (T_2_ plants). Hemizygous or homozygous status was determined based on the amount of GFP-positive haploid pollen (50% or 100%). Plants with 100% GFP-positive pollen grains were considered homozygous. Progeny of homozygous T_2_ plants were again grown on hygromycin selection and pollen grains were imaged to confirm presence of fluorescence (T_3_ plants). In case of SINE3pro::GFP-SINE3, no T_2_ plant with 100% fluorescent pollen could be identified. Eighteen SINE3pro::GFP-SINE3 in *sine3-1* individual transgenic lines were isolated (T_1_ plants). Of those, 9 SINE3pro::GFP-SINE3 in *sine3-1* lines were taken to the T_3_ plant generation. T_3_ progeny from hemizygous T_2_ plants were grown on hygromycin selection, pollen grains were imaged to confirm presence of fluorescence. In both the T_2_ and T_3_ generations, all the plants were hemizygous. Hemizygous plants were used for the GFP imaging experiment shown in Fig. 4.

### Identifying T-DNA insertional mutants

Putative insertional lines were identified using T-DNA Express, an Arabidopsis gene mapping tool created by the Salk Institute Genomic Analysis Laboratory (http://signal.salk.edu/cgi-bin/tdnaexpress). Lines where the T-DNA insertion was predicted to be in an exon or intron were selected and acquired from the Arabidopsis Biological Resource Center (ABRC). Primers used for genotyping were generated from the T-DNA Primer Design tool also created by the Salk Institute Genomic Analysis Laboratory (http://signal.salk.edu/tdnaprimers.2.html) (Supplemental Table 1). The left borders of T-DNA insertion sites were confirmed by sequencing.

### Sequencing to determine the T-DNA insertion site

The DNA fragment between the left border of the T-DNA insert and the 3’ end of the SINE3 gene was cloned using the left border forward primer (BP) and the corresponding *sine3-1* or *sine3-2* reverse primer (RP) (Supplemental Fig. 1a). The DNA fragment was sequenced and a sequence alignment was generated to compare to the SINE3 genomic DNA sequence (Supplemental Fig. 1b).

**Fig. 1:**
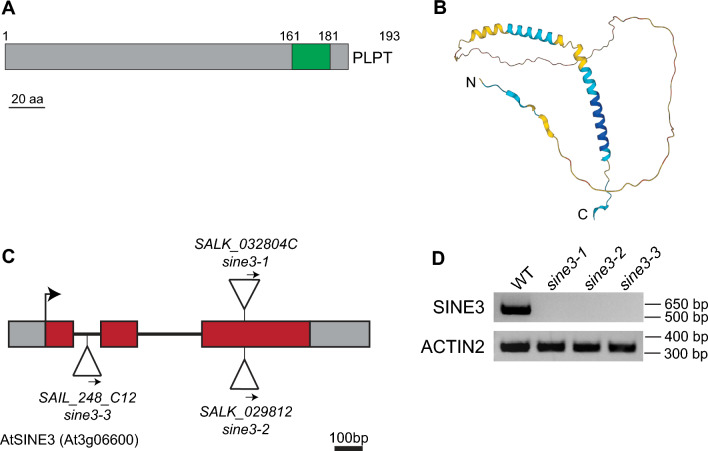
Analysis of *SINE3* mutant alleles. **A** Protein domain organization of SINE3. (Green) transmembrane domain helix; (gray) unknown; (numbers) amino acids. The terminal four amino acids are shown in single-letter code. **B** Predicted tertiary structure of SINE3. The per-residue confidence score between 0 and 100 produced by Alphafold is shown in colors; dark blue > 90, very high; blue > 70, confident; yellow > 50, low; orange < 50, very low. **C** Gene diagram of the SINE3 locus and insertion sites of T-DNA insertional mutants used in this study. The T-DNA insertions in *sine3-1*, *sine3-2*, and *sine3-3* are depicted as triangles, and the arrows indicate the orientation of the T-DNA insert within the chromosome. Exons, red bars; introns, lines; 5’ and 3’ untranslated regions, gray bars. Numbers indicate SALK or SAIL T-DNA insertional mutant collection code. **D** RT-PCR determination of the expression level of full length SINE3 in *sine3* mutants. Primers amplifying the coding region were used for RT-PCR from floral tissue and are listed in Supplemental Table 1

### RT-PCR analysis

Flowers from Arabidopsis plants were ground in liquid nitrogen, and total RNA was extracted using RNeasy Plant Mini kit (QIAGEN). First-strand cDNA was synthesized using SuperScript III First-Strand Synthesis System (Life Technologies) and oligodT as a primer. Primers used for PCR are listed in Supplemental Table 2.

### Pollen grain staining

Pollen viability was determined using a dual fluorescent stain containing propidium iodide and fluorescein diacetate (Hamilton et al. 2015). To visualize nuclei and determine male gametophyte developmental stages, fresh anthers were squashed in 3 µg/mL DAPI in pollen isolation buffer (PIB; 100 mM NaPO_4_, pH 7.5, 1 mM EDTA, and 0.1% [v/v] Triton X-100) and imaged (Backues et al. 2010).

### Floral Staging

Flowers were selected for use in ovule experiments at approximate stages of floral development, as previously described (Alvarez-Buylla et al. 2010).

### Alphafold tertiary structure analysis

The three-dimensional (3-D) structure of the AtSINE3 protein was predicted using the Alphafold Protein Structure Database (https://alphafold.ebi.ac.uk/) (Evans et al. 2021; Jumper et al. 2021; Varadi et al. 2021).

### Ovule development

For the analysis of embryo sac development in wild type and *sine3* mutants, ovules were fixed and cleared as previously described (Min et al. 2019) with a few modifications. Briefly, pistils of floral stage 6 to stage 12 were harvested. For fixation, dissected ovules were incubated in 4% glutaraldehyde (in 12.5 mM K_3_PO_4_, pH 6.9) for 4 h. The tissues were dehydrated through an ethanol series (10, 20, 40, 60, 80, 95, and 100% (v/v) in ddH_2_O) with 10 min per step and left in 100% ethanol overnight. The dehydrated tissues were subsequently cleared in 2:1 (v/v) benzyl benzoate: benzyl alcohol for 5 h, and then observed with a Nikon C2plus confocal laser microscope. Samples were excited with 561 nm wavelength and emission was detected at 566–640 nm.

### Imaging GFP localization in ovules

For imaging GFP-SINE3 subcellular localization in ovules, ovules were fixed and cleared as previously described (Tofanelli et al. 2019). Briefly, pistils were excised from flowers at different developmental stages and fixed in 4% paraformaldehyde in 1X PBS solution for 2 h at room temperature. The fixed pistils were washed twice in 1X PBS for 1 min. Next, the pistils were transferred to 1 mL of ClearSee solution (10% [w/v] xylitol, 15% [w/v] sodium deoxycholate, 25% [w/v] urea) and cleared overnight at room temperature. The cleared pistils were mounted in immersion oil and imaged with a Nikon C2plus confocal laser microscope.

### Aniline blue staining

Aniline blue staining was conducted as previously described (Mori et al. 2006; Wu et al. 2010). Briefly, Arabidopsis pistils from one- or two-day-old flowers after flowering were fixed in a 3:1 ethanol/acetic acid solution for at least 2 h at room temperature. The fixed pistils were then washed in distilled water three times for 5 min each. The pistils were softened in 8 M NaOH overnight at room temperature. Carefully, the softened pistils were washed in distilled water three times for 1 h each and then stained with aniline blue solution (0.1% aniline blue in 0.1 M K_3_PO_4_ buffer, pH 11) for 3 to 5 h in the absence of light. After incubation, stained pistils were carefully mounted and imaged under Nikon C1 confocal laser microscope.

### β-glucuronidase staining

*A. thaliana* seedlings and floral tissue were fixed in 90% acetone on ice for 30 min. Samples were washed in reaction buffer (50 mM sodium phosphate buffer, pH 7.2, 0.1% [vol/vol] Triton X-100, 2 mM K_3_Fe(CN)_6_, 2 mM K_4_Fe(CN)_6_) and incubated in the GUS staining solution (50 mM sodium phosphate buffer, pH 7.2, 0.1% [v/v] Triton X-100, 2 mM K_3_Fe(CN)_6_, 2 mM K_4_Fe(CN)_6_, and 2 mM X-Gluc [GoldBio]) for 48–72 h at 37 °C. Staining solution was then removed, and the samples were washed with 70% ethanol until the tissue was cleared. Samples were imaged under a Nikon SMZ1270 stereo microscope.

### Quartet analysis

The *qrt1-4* mutant was obtained from the Arabidopsis Biological Research Center (SALK_024104, Francis et al. 2006). Homozygous *sine3-1* was crossed with *qrt1-4*. The resulting F1 seeds were grown and allowed to self-fertilize. F2 seeds were screen for homozygosity for both the *sine3-1* and *qrt1-4* alleles via PCR genotyping (See Supplemental Table 1 for primers). In the F3 generation, *sine3-1 qrt1-4* plants were used for Alexander Staining to determine viability in each quartet (Supplemental Fig. 2, Table 3).

## Results

### SINE3 is a plant KASH protein of unknown function

SINE3 was identified previously as a putative Arabidopsis Klarsicht/ANC-1/Syne Homology (KASH) protein (Zhou et al. 2014). SINE3 is a 193 amino acid protein that contains a transmembrane domain (TMD) and KASH tail at its C-terminus (Fig. 1A). The cytoplasmic domain at the N-terminus (amino acids 1–158) is predicted to be highly disordered and has no known domains (Fig. 1B). SINE3 is a plant-specific protein that is not deeply conserved, with homologues only found in the *Brassicaceae* (Poulet et al. 2017).

To identify biological roles of SINE3, we chose three T-DNA insertion mutant alleles, *sine3-1* (SALK_032804C), *sine3-2* (SALK_029812), and *sine3-3* (SAIL_248_C12). Through sequencing, the T-DNA insertion sites were confirmed to be within the third exon for both *sine3-1* and *sine3-2* (Fig. 1C). Although *sine3-1* and *sine3-2* are independent T-DNA insertion lines, the insertion is at the same exact position, between nucleotides 690 and 691 (Supplemental Fig. 1). The parental line for *sine3-2* contained an additional heterozygous T-DNA insertion in the promoter for the gene locus At4g29780, which was removed through segregation, indicating that the two original SALK lines had arisen independently. The T-DNA insertion site for *sine3-3* (determined by the Salk Institute Genomic Analysis Laboratory (Alonso et al. 2003)) was at nucleotide 144 within the first intron (Fig. 1C). RT-PCR analysis revealed that no residual full-length SINE3 transcripts were detected in *sine3-1*, *sine3-2*, and *sine3-3* (Fig. 1D).

### Loss of SINE3 leads to a female-derived seed set defect

**Fig. 2:**
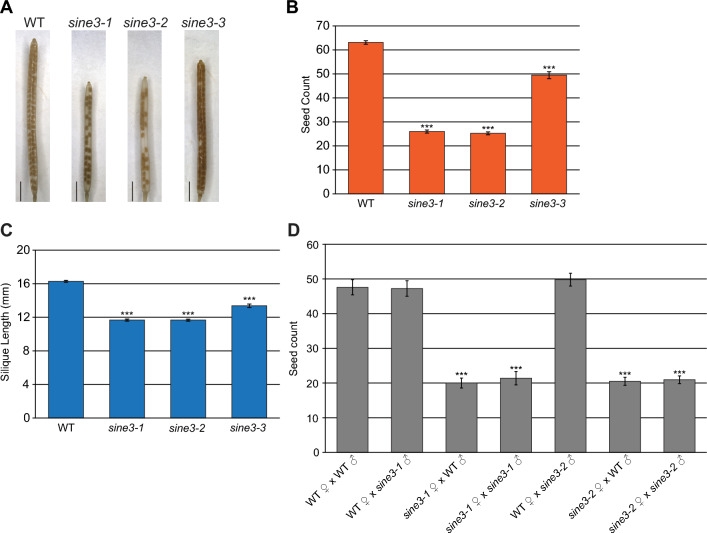
Loss of *SINE3* leads to a seed set defect. **A** Representative images of mature siliques in WT and indicated *sine3* mutants. Scale bar = 2 mm. **B-C** The average number of seeds per silique (**B**) and silique length (**C**) in WT and *sine3* mutants. Data are mean values ± SE (N ≥ 50 siliques per background). Asterisks denote statistical significance to WT, as determined by Student’s *t* test, (*p* < 0.001). **D** Number of seeds per silique after reciprocal crosses between WT and *sine3-1* or between WT and *sine3-2*. Data are mean values ± SE (N ≥ 20 siliques per cross). Asterisks denote statistical significance to WT X WT, as determined by Student’s *t* test, (*p* < 0.001)

When growing homozygote *sine3* mutant plants, a reduction in silique length was noted (Fig. 2A). Quantification showed that both seed number and silique length were significantly reduced. In WT, the average seed number per silique was 63, and the average silique length was 16.3 mm (Fig. 2B, C). In *sine3-1* and *sine3-2*, seeds per silique were reduced to about 25, with siliques measuring on average 11.7 mm (Fig. 2B, C). In contrast, seeds per silique and silique length in *sine3-3* were reduced to 52 seeds and 14.1 mm, respectively, suggesting that *sine3-3* is a weaker allele (Fig. 2B, C). To determine if the seed set defect was derived from the male or female parent, we performed reciprocal crosses between WT and homozygous *sine3-1* and *sine3-2* mutants. When WT stigmas were pollinated with WT, *sine3-1*, or *sine3-2* pollen, the number of seeds per silique was between 48 and 50 (Fig. 2D). In contrast, when either *sine3-1* or *sine3-2* stigmas were pollinated with WT, *sine3-1*, or *sine3-2* pollen, the number of seeds per silique was significantly reduced to approximately 20 (Fig. 2D). This suggests that the reduction in seeds in the two *sine3* mutant alleles was driven by defects related to the female parent.

### *SINE3* is expressed in *Arabidopsis* seedling roots and in reproductive tissues.

**Fig. 3:**
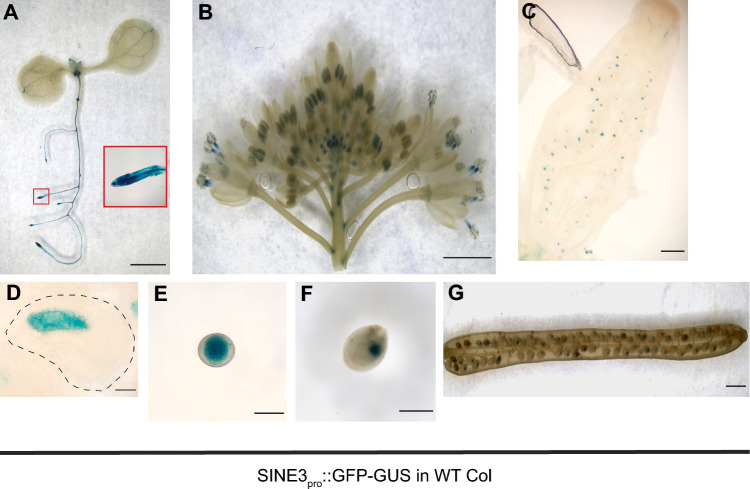
*SINE3* promoter-driven GUS expression in various cell types. **A-G** Expression pattern of *SINE3* revealed by SINE3pro::GFP-GUS transformed WT plants. GUS staining shows the overall expression in an Arabidopsis seedling [the red inset is a magnified image showing lateral root tip expression] (**A**), inflorescence (**B**), pistil (**C**), ovule [black dashed line outlines whole ovule] (**D**), pollen grain (**E**), seed (**F**), silique (**G**). Scale bars = 2 mm (**A-B**); 0.2 mm (**C**); 20µm (**D-E**); 0.25 mm (**F**); 1 mm (**G**)

To examine *SINE3* expression, 2.2. kb of *SINE3* promoter sequence were fused with an in-frame fusion of GFP and β-glucuronidase (GUS) and transformed into WT Arabidopsis plants (SINE3pro::GFP-GUS). In 10 day old SINE3pro::GFP-GUS WT transgenic seedlings, GUS activity was detected throughout the root, the hypocotyl, the shoot apical meristem and faintly in the cotyledon vasculature (Fig. 3A). The strongest signal was detected in and around the root meristems (insert in Fig. 3A). In the inflorescence, a GUS signal was detected in the anthers throughout floral development and in open flowers (Fig. 3B). A GUS signal was observed in unfertilized ovules, specifically in the embryo sac of the ovule, and in pollen grains (Fig. 3C–E). A GUS signal was also detected in siliques, specifically in fertilized ovules and in seeds (Fig. 3F and G). The GUS signal observed is consistent with *SINE3* expression data in the eFP Browser and Arabidopsis Heat Tree Viewer expression databases (Winter et al. 2007; Boavida et al. 2011; Borges et al. 2008; Honys and Twell 2004; Qin et al. 2009; Schmid et al. 2005). In particular, the *SINE3* promoter-driven GUS signal in reproductive development is consistent with publicly available transcriptomic data from these databases.

**Fig. 4:**
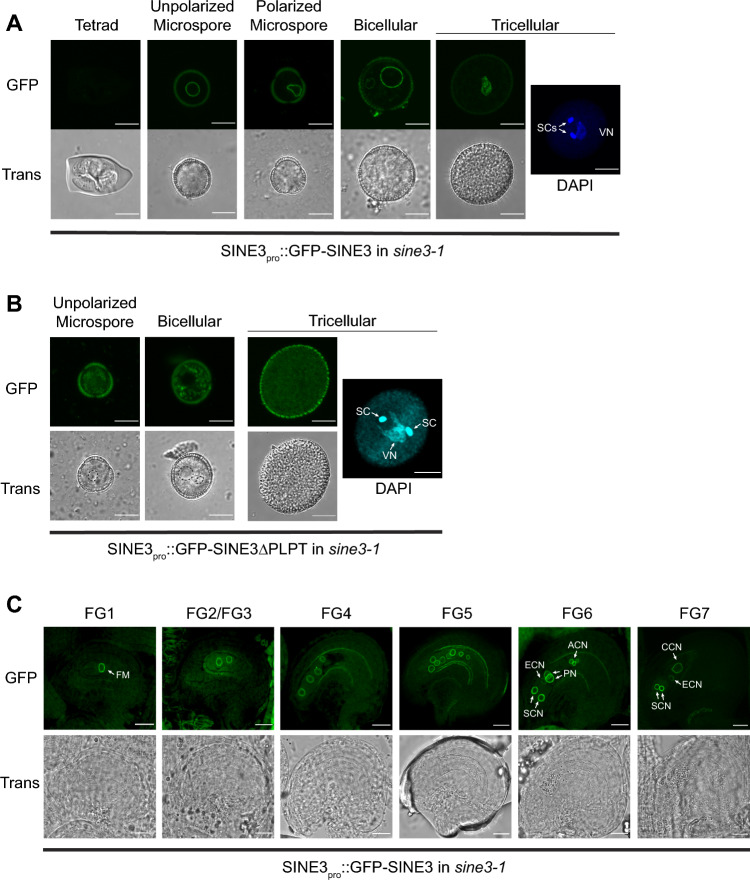
*SINE3* is expressed throughout male and female gametophyte development. **A** Representative microscopic images of the expression and localization of SINE3pro::GFP-SINE3 in *sine3-1* in developing pollen grains at the tetrad, unpolarized microspore, polarized microspore, bicellular, and tricellular pollen stages. Tricellular pollen grain counterstained with DAPI to show the position of the vegetative nucleus (VN) and sperm cells (SCs). Scale bar = 10 μm. **B** Representative microscopic images of the expression and localization of SINE3pro::GFP-SINE3ΔPLPT in *sine3-1* in the unpolarized microspore, bicellular, and tricellular pollen stages. Tricellular pollen grain counterstained with DAPI to show the position of the vegetative nucleus (VN) and sperm cells (SCs). Scale bar = 10 μm. **C** Images of developing ovules at stages FG1 to FG7. FM, functional megaspore; *ACN*, antipodal cell nucleus; *SCN*, synergid cell nucleus; *ECN*, egg cell nucleus; *CCN*, central cell nucleus. All images were captured using confocal microscopy. Scale bar = 20 μm

Next, hemizygous SINE3pro::GFP-SINE3 in *sine3-1* transgenic plants (see Methods) were utilized to examine SINE3 expression and subcellular localization during gametophyte development. We first analyzed SINE3pro::GFP-SINE3 in *sine3-1* during pollen development (Fig. 4A). GFP was not detected at the tetrad stage but was present at the NE in unpolarized and polarized microspores, bicellular pollen, and tricellular pollen. In bicellular pollen, the GFP-SINE3 signal was stronger at the vegetative NE than at the generative NE. In mature pollen grains, the GFP-SINE3 signal was only detectable at the vegetative NE (Fig. 4A).

In the sporophyte, we have shown that the association of GFP-SINE3 with the nuclear envelope depends on the most C-terminal four amino acids (PLPT), which are required for binding SUN proteins (Zhou et al. 2014). To assess if the association with the gametophytic NE followed the same requirements, we also expressed the truncated SINE3pro::GFP-SINE3ΔPLPT in *sine3-1.* As shown in Fig. 4b, this led to diffuse GFP fluorescence in unpolarized microspores and at the bicellular and tricellular stage, indicating that the mechanism of SINE3 association with the nuclear envelope is the same in the sporophyte and gametophyte.

When GFP-SINE3 expression and localization was determined during female gametophyte development, GFP-SINE3 was detected at the NEs in ovules at all developmental stages (Female Gametophyte 1 (FG1) through FG7; Fig. 4C). The GFP-SINE3 fluorescent signal was detected on nuclear envelopes across all developmental stages. Together, these data show that the SINE3 promoter is specifically active in both male and female gametophytes, that SINE3 is expressed during all stages of gametophyte development, and that the protein is associated with the nuclear envelope at all stages, dependent on the last four amino acids. In pollen, nuclear envelope localization of SINE3 is limited to the vegetative cell.

### *sine3* mutant ovules arrest at the FG1 stage of female gametophyte development

**Fig. 5:**
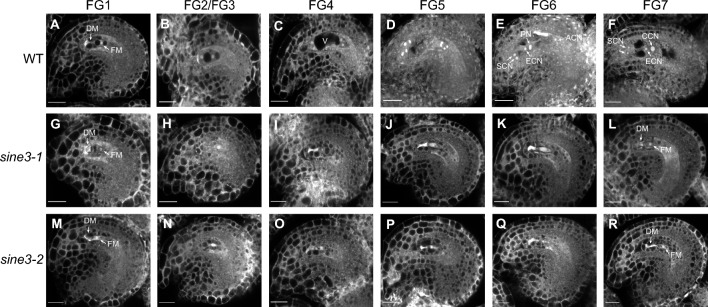
Loss of *SINE3* leads to defects in female gametophyte development. Ovules at different developmental stages in WT (**A-F**), *sine3-1* (**G-L**), and *sine3-2* (**M-R**): FG1 (**A, G, M**), FG2/3 (**B, H, N**), FG4 (**C, I, O**), FG5 (**D, J, P**), FG6 (**E, K, Q**), FG7 (**E, L, R**). *DM*, degenerated megaspore; *FM*, functional megaspore; *ECN*, egg cell nucleus; *PN*, polar nucleus; *ACN*, antipodal cell nucleus; *CCN*, central cell nucleus; *SCN*, synergid cell nucleus; *V*, vacuole. Bright white spots indicate nucleoli. Scale bar = 20 µm

Because the seed set defect in *sine3* mutants was driven by a defect from the female, we observed WT, *sine3-1*, and *sine3-2* ovules at various stages of female gametophyte development using whole-mount tissue clearing (Min et al. 2019). In WT, female gametogenesis proceeded normally from female gametophyte stage 1 (FG1) to FG7 (Fig. 5A–F). WT ovules at FG1 contained one functional megaspore (FM) which then underwent three rounds of mitosis without cytokinesis resulting in eight nuclei (Fig. 5A–D; FG2-FG5). Following the subsequent nuclear fusion of two polar nuclei and the degeneration of the three antipodal cells (Fig. 5E; FG6), WT ovules reached maturity (Fig. 5F; FG7). In the *sine3-1* and *sine3-2* mutants, female gametogenesis proceeded normally in approximately half of the ovules, however, the other half appeared to arrest at FG1 (Fig. 5G–L for *sine3-1* and Fig. 5M–R for *sine3-2*). When analyzing the female gametophytes at the mature stage in WT, *sine3-1*, and *sine3-2,* 95.4% of WT gametophytes reached the FG7 stage (Fig. 5F; Table 1). By contrast, only 47% of the *sine3-1* ovules and 47.8% of the *sine3-2* ovules reached the FG7 stage, while 51.4% of *sine3-1* and 51% of *sine3-2* ovules were arrested at FG1 (Fig. 5L, R; Table 1). Taken together, these results indicate that SINE3 plays an important role at the onset of the nuclear divisions during female gametophyte development and that the mutant phenotype has an about 50% penetrance.

**Table 1:** Developing *sine3* mutant ovules arrest at female gametophyte stage (FG) 1

Genotype	FG1 (%)	FG2 (%)	FG3 (%)	FG4 (%)	FG5 (%)	FG6 (%)	FG7 (%)	*n*
WT	1	0	0	0.3	1	2.3	95.4	306
*sine3-1*	51.4	0	0	0	0.6	1	47	315
*sine3-2*	51	0.3	0.3	0	0	0.6	47.8	335

### Loss of SINE3 leads to a pollen viability defect

**Fig. 6:**
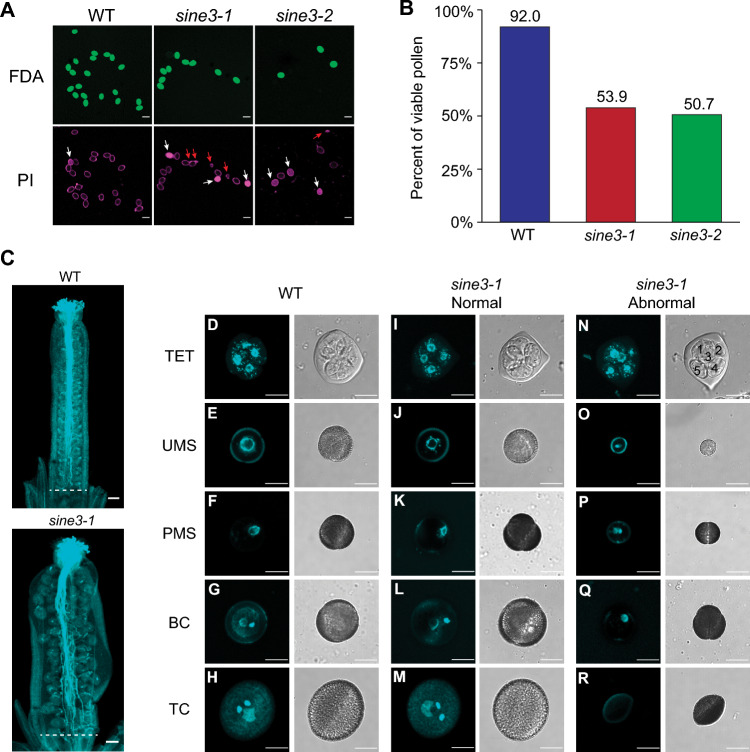
Loss of *SINE3* perturbs pollen development and leads to defects in viability. **A-B** Representative images (**A**) and quantification of viability (**B**) of WT and *sine3* mutant pollen after hydration. Pollen was incubated for 30 min in pollen germination media containing fluorescein diacetate (FDA) and propidium iodide (PI), dyes that stain viable and non-viable pollen, respectively. White arrows mark non-viable normal-size pollen grains and red arrows mark non-viable shriveled or collapsed pollen grains. N > 1260 pollen grains per background. Scale bar = 25 μm. (**C**) Aniline blue-stained pollen tubes in self-pollinated WT and *sine3-1* pistils. The white dotted lines indicate the pollen tube growth front in the pistils. Scale bar = 100 µm. **(D)** Developing spores and pollen grains stained with DAPI from WT (**D-H**) and *sine3-1* (**I-R**) plants at tetrad (TET; **D, I, N**), unpolarized microspore (UMS; **E, J, O**), polarized microspore (PMS; **F, K, P**), bicellular (BC; **G, L Q**), and tricellular (TC; **H, M, R**) pollen stages. Developing *sine3-1* pollen grains were split into 2 groups: pollen exhibiting WT-like development (**I-M**) and pollen exhibiting abnormal development (**N-R**). Images in each column correspond to the stages indicated above, with the exception of P and Q, which were present at the UMS, PMS, and BC stages

Approximately half of the genes identified as functioning in gametogenesis are required for both female and male gametophyte development (Pagnussat et al. 2005). Thus, we next determined if the loss of SINE3 also affected the male gametophyte. Using a dual fluorescent stain containing propidium iodide and fluorescein diacetate (Hamilton et al. 2015), we observed a reduction in pollen viability in *sine3-1* and *sine3-2* mutants. Many of the nonviable pollen grains in *sine3-1* and *sine3-2* mutants were shriveled or collapsed (Fig. 6A, red arrows; white arrows mark non-viable normal-size pollen grains). 53.9% of *sine3-1* pollen grains (*n* = 1264) and 50.7% of *sine3-2* pollen grains (*n* = 1269) were viable, compared to 92% of WT pollen grains (*n* = 1310) (Fig. 6B).

We then outcrossed the homozygous *sine3-1* mutant to a *quartet1* (*qrt1-4*) mutant (Francis et al. 2006). In *qrt1-4* plants, the four products of a single meiosis remain attached to each other throughout pollen development. Alexander staining showed that the *qrt1-4* plant produced four attached yet otherwise normal mature pollen grains (Supplemental Fig. 2). However, between zero and four shriveled and nonviable pollen grains were detected in tetrads from the F3 *sine3-1 qrt1-4* homozygous double mutant plants at an about equal distribution (Supplemental Fig. 2 and Supplemental Table 3), suggesting that the male defect occurs with about 50% penetrance and occurs randomly after meiosis. These data also show that the gametophyte development phenotype is linked to the *sine3-1* allele, which was followed in this cross by PCR detection of the T-DNA insertion to the F3 generation.

When self-pollinated Arabidopsis pistils from WT and *sine3-1* were fixed and stained with aniline blue to observe pollen tube growth within the transmitting tract, abundant *sine3-1* pollen tubes germinated and grew successfully through the stigma and the pollen tube transmitting tract, and neared the ovules throughout the pistil, similar to WT (Fig. 6C). This indicates that the viable *sine3-1* pollen grew normally and that pollen tube growth and guidance were normal in *sine3-1* pistils.

Together, these data suggest that loss of SINE3 also perturbs male gametophyte development. Like the female gametophyte defect, the male defect occurs with about 50% penetrance and occurs randomly after meiosis. This defect had no effect on the male reciprocal cross (see Fig. 2D), likely because viable pollen was present in abundance.

### *sine3* mutant microspores arrest prior to pollen mitosis I

Based on the significant decrease in pollen viability and the collapsed pollen phenotype observed at the mature pollen stage, we wanted to determine when the initial defect appears during pollen development. We thus examined DAPI-stained microspores dissected from staged anthers of WT and *sine3-1* mutant plants (Fig. 6D-R; Table 2). A typical progression of pollen development was observed in WT plants (Fig. 6D-H; Table 2). Meiosis resulted in a tetrad of 4 equally sized microspores (Fig. 6D). Upon callose degradation, the microspores were released from the tetrad (Fig. 6E). The microspore became polarized with the migration of the nucleus to the germ cell pole (Fig. 6F). The polarized microspore underwent an asymmetric mitotic division (pollen mitosis I, PMI) to form the germ cell nucleus and vegetative nucleus (Fig. 6G). A second symmetric mitotic division (pollen mitosis II, PMII) of the generative cell nucleus generated two sperm cells (Fig. 6H).

**Table 2:** Pollen developmental analysis in WT and *sine3-1* mutant plants

	Tetrad (TET)		Unpolarized microspore (UMS)		Polarized microspore (PMS)		Bicellular (BC)		Tricellular (TC)	
	WT	*sine3-1*	WT	*sine3-1*	WT	*sine3-1*	WT	*sine3-1*	WT	*sine3-1*
TET	152	140								
UMS			149	139	5	18		9		
PMS			3	3	149	123	1	51		
BC							149	94		4
TC									150	107
AbTET (5)		9								
AbTET (6)		4								
AbUMS				13		14		14		
AbDEG									1	87
Abnormal (%)	0	8.5	2.0	8.4	3.2	9.0	1.0	44	1.0	46
Total (n)	152	153	152	155	154	155	150	168	151	198

Expected developmental stages are listed across. Stages and abnormalities as detected are listed in the left column

*TET*, tetrad; *UMS*, unpolarized microspore; *PMS*, polarized microspore; *BC*, bicellular pollen; *TC*, tricellular pollen; AbTET (5), abnormal tetrad containing 5 nuclei; AbTET (6), abnormal tetrad containing 6 nuclei; AbUMS, abnormal phenotype resembling smaller unpolarized microspore pollen; AbDEG, abnormally degenerating pollen with or without residual DAPI staining

In *sine3-1* plants, aberrant phenotypes were first evident at the tetrad stage (Fig. 6I and N). WT had 100% normal tetrads composed of four equally sized microspores (Fig. 6D). In *sine3-1*, the majority of tetrads (91.5%) were also normal (Fig. 6I; Table 2) However 8.5% of *sine3-1* tetrads contained 5 or 6 spores that were not equally sized (Fig. 6N; Table 2). At later developmental stages, a population of abnormally small microspores was observed in *sine3-1* (Fig. 6O, P, Table 2), in addition to normal microspores (Fig. 6J; Table 2). Nuclear migration towards the future germ cell pole occurred in *sine3-1* mutant microspores but appeared slightly delayed (Fig. 6K, Q; Table 2). Only 56% of microspores underwent PMI to form bicellular pollen (Fig. 6L; Table 2), with a large proportion of microspores remaining polarized (Fig. 6Q; Table 2). The WT-like *sine3-1* bicellular pollen underwent PMII to form tricellular pollen containing a vegetative nucleus and 2 sperm cells (Fig. 6M; Table 2). The proportion of aberrantly small microspores, and unpolarized and polarized microspores observed at the bicellular pollen stage accounts roughly for the proportion of collapsed pollen grains observed at the mature pollen stage (44%) (Fig. 6R; Table 2).

Together, these data show that SINE3 is also required for male gametophyte development and that the mutation leads to a variety of defects. Most prominent is the inability of mutant pollen to progress from the polarized microspore to the bicellular pollen stage, in other words to complete pollen mitosis I. Like in the female, this phenotype has about 50% penetrance, with remaining microspores developing to fully mature, viable pollen grains capable of pollen tube growth, female signal perception, and fertilization.

## Discussion

In this study, we have shown that the plant KASH protein SINE3 plays an important role in plant gametophyte development. Both male and female gametophyte development are defective in *sine3* mutants. Approximately half of the male gametophytes of *sine3* were arrested at the polarized microspore stage prior to pollen mitosis I. Less than 50% of *sine3* female gametophytes completed the three rounds of mitosis to form mature female gametes. The remaining ovules were arrested at the FG1 developmental stage, prior to the first post-meiotic mitosis. SINE3 was expressed in male and female gametophytes and located at the nuclear envelope (NE) at all developmental stages. Together, these data suggest that SINE3 is important for the initiation of the first post-meiotic nuclear/cell division in both female and male gametophytes. The incomplete penetrance of the mutant phenotype observed here has been reported in other reproductive mutants, indicating that this is not an unusual phenomenon among Arabidopsis reproductive mutants (Chen and McCormick 1996; Howden et al. 1998; Park et al. 1998).

Previous studies have dissected the mechanisms of male and female gametophyte development through mutant identification and characterization. In *gemini pollen 1* (*gem1*) mutant microspores, nuclear migration towards the future germ cell pole was impaired, thus resulting in similar daughter cells and failed germ cell differentiation (Park et al. 1998). *gem1* was later identified as an allele of *MOR1*, which is a member of the microtubule-associated protein (MAP) 215 family (Whittington et al. 2001; Twell et al. 2002; Oh et al. 2010b). In microspores of sidecar pollen (*scp*) mutants, nuclear migration occurs normally, but a proportion of pollen contains an extra vegetative cell, attributed to a delay in nuclear division and an altered spindle orientation (Chen and McCormick 1996; Oh et al. 2010c, 2011). Unlike *gem1* and *scp* mutants, defective *sine3* mutant microspores did not undergo division and the nucleus remained polarized.

A plethora of other female gametophyte mutants that affect cell cycle progression have been identified and many have a similar phenotype to *sine3* mutants (Elliott et al. 1996; Baker et al., 1997; Christensen et al. 1998; Springer et al. 2000; Acosta-Garcia and Vielle-Calzada 2004; Huanca-Mamani et al. 2005; Colombo et al. 2008; Latrasse et al. 2008; Liu et al. 2008; Gallois et al. 2009; Backues et al. 2010; Sankaranarayanan et al. 2020; Qin et al. 2022). These mutants affect fundamental cellular processes, such as gene expression and regulation (Elliott et al. 1996; Baker et al., 1997; Huanca-Mamani et al. 2005; Colombo et al. 2008; Latrasse et al. 2008), DNA replication (Springer et al. 2000), and protein degradation (Liu et al. 2008; Gallois et al. 2009).

A mutation with similar characteristics to *sine3-1* and *sine3-2* is *mos7-5*, a mutant allele of the nucleoporin MOS7 (Modifier of Snc1, 7), the Arabidopsis homolog of Nup88 (Park et al. 2014). Nuclear migration in *mos7-5* mutant microspores occurs normally but arrests at pollen mitosis I, similar to *sine3* mutants. Loss of MOS7 also affected female gametophyte development, similar to *sine3* mutants, with developing female gametophytes arrested at FG1 (Park et al. 2014). MOS7 was shown to be required for spindle assembly during pollen mitosis I and is localized at the spindle during mitosis (Park et al. 2014).

Like MOS7, SINE3 is a NE-associated protein, currently of unknown function in reproductive development. SINE3 is a plant KASH protein, the ONM component of plant LINC complexes (Zhou et al 2014). LINC complexes, which facilitate nuclear movement and nuclear positioning, span the nuclear envelope, with KASH proteins frequently interfacing directly and indirectly with the cytoskeleton (reviewed in Meier et al. 2017). At this point, it is not known at which step of the male and female gametophyte cell cycles the *SINE3* gene product acts. However, based on its nuclear envelope localization and its dual role in both gametophytes, one attractive hypothesis is that the protein is directly involved in a process required for the initiation of the first post-meiotic mitosis. In land plants, that do not have centrosomes, the nuclear envelope is involved in forming the microtubule organizing centers (MTOCs) for the outgrowth of the spindle apparatus at the onset of prophase (Zhang and Dawe 2011; Masoud et al. 2013). Further work is required to investigate this step specifically, e.g. by imaging fluorescently labeled microtubules in *sine3* mutants during pollen mitosis I (Oh et al. 2010a).

*Sine3* mutants also displayed a minor male meiosis defect, with approximately 8.5% of tetrads containing more than four spores (Table 2). In animals, LINC complexes have been shown to be involved in meiosis, in particular the KASH protein KASH5, which associates with Dynein (Morimoto et al. 2012; Horn et al. 2013; Agrawal et al. 2022). In plants, a KASH protein that functions during meiosis has not yet been identified. However, there is a precedent for a LINC-complex role in plant meiosis because in *Oryza sativa* and Arabidopsis, double mutants of *SUN1* and *SUN2* have severe meiotic defects, such as delayed meiotic progression, an absence of full synapsis, unresolved interlock-like structures, and a reduction in the mean cell chiasma frequency (Zhang et al. 2020; Varas et al. 2015). The meiotic defect reported here suggests that SINE3 too plays a minor role during meiosis.

Unlike many other gametophyte mutants, the function of SINE3 appears to be specific to the gametophyte. SINE3 is expressed in sporophytic tissues, such as the root and shoot apical meristems, but the homozygous *sine3* mutants did not exhibit obvious growth defects. One hypothesis is that the presence of other proteins, which are expressed in the sporophyte but not the gametophyte, are acting redundantly to SINE3. Alternatively, the currently unknown molecular role of SINE3 might point towards a step required for the onset of gametophytic, but not sporophytic mitosis. For example, pollen mitosis I is an asymmetric division that produces a cell within a cell and occurs in the absence of a preprophase band (Terasaka and Niitsu 1995; Oh et al. 2010b, 2010c). Female gametophyte mitosis occurs in the absence of cytokinesis, producing a coenocyte. Thus, important regulatory or mechanistic steps might be unique to gametophyte mitosis. Investigating the molecular roles of SINE3 might shed light on such a mechanism.

A limitation of this study was the inability to recover any *sine3-1* transgenic lines that were homozygous for SINE3pro::GFP-SINE3 or SINE3pro::GFP-SINE3ΔPLPT. Eighteen SINE3pro::GFP-SINE3 in *sine3-1* and 13 SINE3pro::GFP-SINE3ΔPLPT in *sine3-1* individual transgenic lines were isolated. Of those lines, 9 SINE3pro::GFP-SINE3 in *sine3-1* and 3 SINE3pro::GFP-SINE3ΔPLPT in *sine3-1* lines were further characterized. In both the T_2_ and T_3_ generations, all plants were hemizygous for the GFP-fusion construct. The inability to isolate a homozygous line seems to suggest that two copies of either SINE3pro::GFP-SINE3 or SINE3pro::GFP-SINE3ΔPLPT are detrimental. Possibly a dose-dependent dominant-negative or neomorphic effect of the fusion proteins is at play. Despite this limitation the two independently isolated mutant alleles *sine3-1* and *sine3-2* as well as the weaker allele *sine3-3* (5’ intron insertion) all recapitulate the same phenotype. *Sine3-1* was outcrossed from two, *sine3-2* from one additional T-DNA insertion present in the original germplasm. *Sine3-1* was additionally crossed with the *qrt1-4* mutant. This strongly suggests that the disruption of the *SINE3* open reading frame is the cause of the mutant phenotypes of the three lines.

Taken together, we provide evidence that the plant KASH protein SINE3 is involved in male and female gametophyte development, as related to male meiosis and male and female mitosis. Further examination of *sine3* mutant defects in meiosis and mitosis will shed light on the molecular function of SINE3 and its potential interactors.

### Author contribution statement

IM, NRG, and MM designed the experiments. MM performed and analyzed the experiments. MM, NRG, and IM wrote and edited the manuscript and IM provided oversight and funding for the study.

## Supplementary Information

Below is the link to the electronic supplementary material.

Supplementary file1 (PDF 684 KB)

## Data Availability

All original data files will be made available upon request.
