# Supplementary material for: The Arabidopsis KASH protein SINE3 is involved in male and female gametogenesis
Source: Plant Reprod. 2024 Sep 16;37(4):521–34. doi: 10.1007/s00497-024-00508-8 (PMC11511747; doi:10.1007/s00497-024-00508-8)
Supplement: Supplementary file 1 — Supplementary file1 (PDF 684 KB) [file 497_2024_508_MOESM1_ESM.pdf]

Supplemental Figures and Tables

The Arabidopsis KASH protein SINE3 is involved in male and female gametogenesis.

Morgan Moser[1,#], Norman R. Groves[1], and Iris Meier[1,2*]

[1]Department of Molecular Genetics, The Ohio State University, Columbus, OH, USA

[2] Center for RNA Biology, The Ohio State University, Columbus, OH, USA

* Address correspondence to meier.56@osu.edu

#Current address: Institute of Genomic Medicine, Nationwide Children's Hospital, Columbus, OH, USA

Supplemental Figure 1

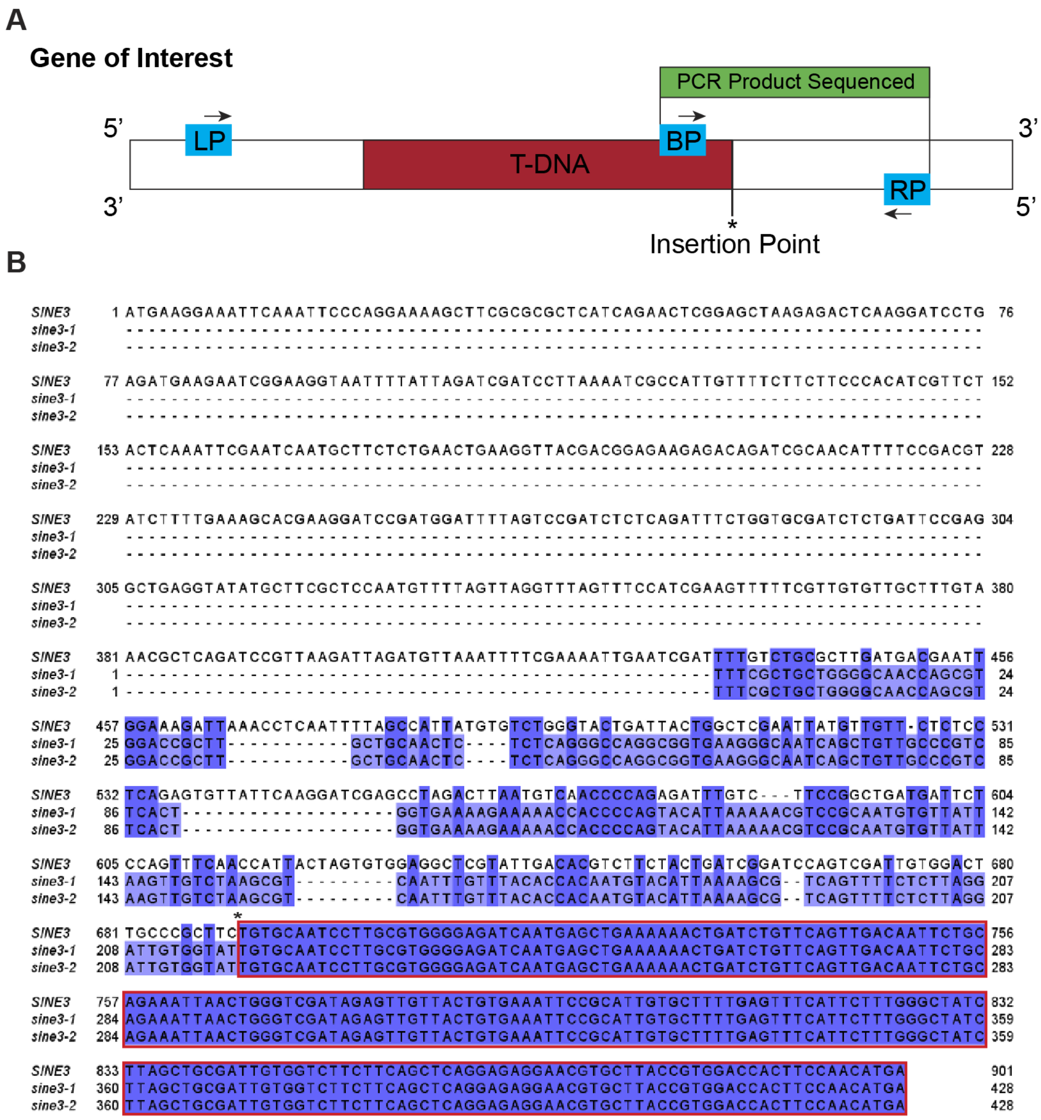

Supplemental Figure 1. Sequencing of *SINE3* T-DNA Insertional lines

(**a**) The gene of interest is shown as open bar, representing the double-stranded DNA. The T-DNA insertion is represented in red and the sequenced PCR product is represented in green. LP and RP are the left and right genomic primers, respectively. BP is the left T-DNA border primer (LB, see http://signal.salk.edu/tdnaprimers.2.html). Arrows above the primers indicate 5' to 3' direction. The point of the T-DNA insert directly adjacent to the genomic DNA is the insertion point (indicated by an asterisk). (**b**) Sequence reads generated for *sine3-1* and *sine3-2* aligned

with the *SINE3* genomic DNA sequence. The asterisks indicate the location of the T-DNA insertion point shown in (a), between the nucleotides 690 and 691 of *SINE3*. The red box indicates identity between the three sequences.

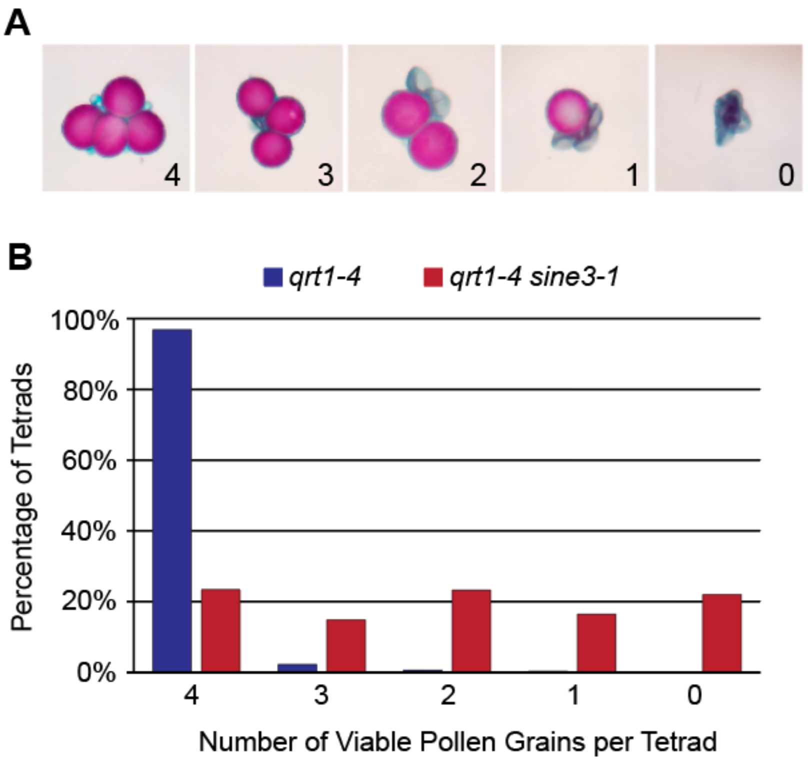

Supplemental Figure 2. Quartet analysis of WT and *sine3-1*.

(A) Images of Alexander staining of the pollen in a homozygous *qrt1-4* background. [4], a tetrad of four normal pollen grains; [3], a tetrad of three viable and one aborted pollen grains; [2], a tetrad of two viable and two aborted pollen grains; [1], a tetrad of one viable and three aborted pollen grains; [0], all aborted tetrad. (B) Percentage of tetrads containing 4, 3, 2, 1 or 0 normal pollen grains in WT versus *sine3-1* mutant. N > 660 tetrads per background.

Primer Pair	Purpose
5'-GGCTCGTATTGACACGTCTTCTACT-3'<br>5'-CTGCTTATGAGGCCTACTTGCA-3'	Amplifying the wild-type sequence of SINE3 for *sine3-1* genotyping
5’-ATTTTGCCGATTTCGGAAC-3’<br>5'-CTGCTTATGAGGCCTACTTGCA-3'	Amplifying the T-DNA sequence of *sine3-1* for *sine3-1* genotyping
5’-CAAATTCCCAGGAAAAGCTTC-3’<br>5’-CCTTTGGTAGACACATCCCAG-3’	Amplifying the wild-type sequence of SINE3 for *sine3-2* genotyping
5’-ATTTTGCCGATTTCGGAAC-3’<br>5’-CCTTTGGTAGACACATCCCAG-3’	Amplifying the T-DNA sequence of *sine3-2* for *sine3-2* genotyping
5’-GAACACGAGAGGAATCGAGC-3’<br>5’-AACTGGAGAATCATCAGCCG-3’	Amplifying the wild-type sequence of SINE3 for *sine3-3* genotyping
5’-GCCTTTTCAGAAATGGATAAATAGCCTTGCTTCC-3’<br>5’-AACTGGAGAATCATCAGCCG-3’	Amplifying the T-DNA sequence of *sine3-3* for *sine3-3* genotyping
5’-TCTCTTCCCAGAAAAGGCTTC-3’<br>5’-CGTGGGTCTCAAGAATCTTTG-3’	Amplifying the wild-type sequence of QUARTET for *qrt1-4* genotyping
5’-ATTTTGCCGATTTCGGAAC-3’<br>5’-CGTGGGTCTCAAGAATCTTTG-3’	Amplifying the T-DNA sequence of *qrt1-4* for *qrt1-4* genotyping
5’-ATGAAGGAAATTCAAATTCCCAG-3’<br>5’-TCATGTTGGAAGTGGTCCACG-3’	RT-PCR analysis of full-length SINE3 transcripts
5’-CTAAGCTCTCAAGATCAAAGGCTTA-3’<br>5’-TTAACATTGCAAAGAGTTTCAAGGT-3’	RT-PCR analysis of the transcripts of the internal reference gene ACTIN2 (At3g18780)

Supplemental Table 1. Primers used for genotyping mutants.

LP and RP primers were generated from the T-DNA Primer Design tool created by the Salk Institute Genomic Analysis Laboratory (http://signal.salk.edu/tdnaprimers.2.html; Suppl. Fig 1.)

Primer Name	Direction	Sequence (5’-3’)	Used to Clone
SINE3Pro2.2kb SacITopoF	Forward	**CACC**<u>GAGCTC</u>AAATTATGGGAGTTGTGTTGT	SINE3 promoter
SINE3Pro SpeITopoR	Reverse	<u>ACTAGT</u>CTCTCTCCAATGTTTTCAAATTTGG	SINE3 promoter
SINE3F	Forward	ATGAAGGAAATTCAAATTCCCAG	SINE3
SINE3R	Reverse	TCATGTTGGAAGTGGTCCACG	SINE3
SINE3dPLPTR	Reverse	TCATCCACGGTAAGCACGTTCCTCT	SINE3 ΔPLPT
GUSF	Forward	**CACC**ATGTTACGTCCTGTAGAAACCCCAA	GUS
GUSR	Reverse	TCATTGTTTGCCTCCCTGCTGCGGT	GUS

Supplemental Table 2. Primers used for cloning.

CACC sites for directional TOPO cloning are indicated in bold. Specific recognition sites for *SacI* and *SpeI* are underlined.

Background	Viable (%)	Not Viable (%)	*n* (pollen grains)
qrt1-4	2631 (98.9)	29 (1.1)	2660
qrt1-4 sine3-1	1383 (50.3)	1365 (49.7)	2748

Supplemental Table 3. Number of viable and non-viable pollen grains scored in quartet analysis.
